# The Correlation between Intraorifice Distance and the Anatomical Characteristics of the Second Mesiobuccal Canal of Maxillary Molars: A CBCT Study

**DOI:** 10.1155/2024/6636637

**Published:** 2024-01-29

**Authors:** Isabella Perondi, Silvio Taschieri, Martino Baruffaldi, Roberto Fornara, Luca Francetti, Stefano Corbella

**Affiliations:** ^1^Department of Biomedical Surgical and Dental Sciences, Università degli Studi di Milano, Milan, Italy; ^2^IRCCS Ospedale Galeazzi Sant'Ambrogio, Milan, Italy; ^3^Department of Oral Surgery of the Institute of Dentistry, I.M. Sechenov First Moscow State Medical University (Sechenov University), Moscow, Russia; ^4^Private Practice in Piacenza, Piacenza, Italy; ^5^Private Practice in Marcallo con Casone (MI), Milan, Italy

## Abstract

**Introduction:**

Missing anatomy is one of the main causes of endodontic failures, and accurate knowledge of teeth anatomy is a prerequisite for adequate root canal treatment. The aim of the present cone beam computed tomography (CBCT) study was to describe the anatomical characteristics of the mesiobuccal (MB) root canals of maxillary molars and to understand if a correlation exists between the position of the canal orifices and the anatomical features of the root.

**Methods:**

For the purposes of the study, a total of 100 CBCT scans of maxillary molars with two MB canals were selected and studied. The features of root canal anatomy of the MB root of the same teeth were analyzed and recorded (root length, confluence, and Vertucci classification). The distance between MB1 and MB2 orifices and the palatal orifice were recorded, as well as the distance between the orifices and the line joining the palatal orifice and the others. A statistical analysis was performed by providing descriptive measures, the measure of the correlation between different parameters, and the influence of some of these measures on the presence of a confluence between MB1 and MB2.

**Results:**

It resulted that the most frequent configuration is type II Vertucci. The length measured on the sagittal plane was significantly correlated to the presence of a confluence in the MB root. When the root length was higher than 14.56 mm, the confluence is three times more frequent than when the length is lower (OR = 3.635). The area under the curve (AUC) of the receiver operator characteristic (ROC) curve for length on the sagittal plane was 0.632 (*P*=0.036).

**Conclusions:**

The presence of a confluence between the MB canals of maxillary molars is correlated to the length of the root that could be measured on the sagittal plane and to the distance between the canal orifices. The relative position of the root canal orifices in relation to anatomic landmarks needs to be further explored.

## 1. Introduction

Primary root canal therapy was associated to relatively high success rate over time (ranging from 82.0% to 92.6% depending on the criteria adapted in the reference studies), as reported in one recent systematic review of the literature [1].

Missed anatomy, that may cause the entire portions of the root canal left untreated, is one of the most important causes of failure of the root canal treatment because of the impossibility of removing all the pulpal debris [2]. One recently published paper on a total of 633 maxillary molars examined through cone beam computed tomography (CBCT) found that in 43.6% of cases a periapical lesion was associated to missed anatomy and, of these cases, 81.7% showed that the missed canal was the second mesiobuccal (MB2) canal of maxillary molars [3]. Similar results were published in 2020 by Baruwa et al. [4] who found that, on a huge sample of more than 20,000 teeth, considering just the ones with one missed canal, 82.6% presented a periapical lesion; moreover, the MB root of the maxillary first molar was the root that most frequently presented a missed canal [4]. Such results confirmed the paramount importance of detecting and treating the entire root canal system to lower the risk of endodontic treatment failure [5, 6].

In general terms, the precise identification of the root canal system anatomy while performing endodontic treatment could be complicated by the presence of aberrant or unusual canal configurations, accessory canals, bifurcations, isthmuses, canal anasthomosis, as well as canal confluences [7]. Due to the known variability of maxillary first molar root canal anatomy, the precise identification of MB2 canal should be considered fundamental for creating the conditions for long-term success of endodontic treatment [8].

The CBCT technology was independently developed by two study groups with the aim of producing tridimensional imaging of the oral structures while reducing the dose of radiation as compared to conventional computed tomography [9, 10]. In endodontics, the applications of CBCT for diagnostic purposes are indicated in the joint position paper of the American Academy of Endodontics and American Academy of Oral and Maxillofacial Radiology updated in 2015 and by the guidelines of European Society of Endodontics published in 2015 [11, 12]. Both papers clearly affirmed that the use of CBCT could be indicated in the cases of unusual or complex root canal anatomy that could be hypothesized after bidimensional periapical radiographs 2015 [11, 12].

The evaluation of the accuracy of CBCT for the detection of root canal anatomy and for the detection of MB2 was the objective of a number of studies, examining under different settings and conditions [13–15]. One systematic review of the literature (both on *in vivo* and on *ex vivo* studies) detected a high specificity and sensitivity of the method (more than 90%), being informative for the exploration of the anatomy of the MB root of maxillary first molars [13].

The aim of the present study was, by exploring the anatomic characteristics of MB root of maxillary first molars with two MB canals, using CBCT images, to investigate if correlations exist between the position of canal orifices and the root canal anatomy, in relation to the confluence or not of the two MB canals.

## 2. Materials and Methods

The study was performed using anonymized radiographic records present in the database of the Dental Clinic of the IRCCS Istituto Ortopedico Galeazzi in Milan, Italy. The study protocol was performed in the context of the project (L2053) approved by the scientific board of the institute. After the screening of 252 radiographic records, a total of 100 teeth (maxillary first molars) belonging to 68 subjects were considered. All the radiographs were performed for purposes that were different from the aim of the study (e.g., implant placement, third molar removal, and need of performing other types of surgery), but the subjects provided the informed consent for the use of material, in fully anonymized manner, for research purposes, eventually.

To be included in the study, the radiographs should refer to: (a) teeth (maxillary first molars) presenting both MB1 and MB2 orifices, without any root canal treatment; (b) teeth without any sign of periapical lesion; (c) teeth without any metallic restoration; (d) teeth without signs of root fracture or resorption; (e) teeth clearly presenting the orifice of the MB2; (f) subjects being 16 years old or more; (g) who provided their written informed consent for using their records for research purposes in anonymized form; and (h) CBCT images of good quality, having a voxel size of no more than 0.125 mm.

### 2.1. CBCT and Image Analysis Procedure

The CBCT scans were performed using two devices (3D Accuitomo 170—J. Morita Corporation, Osaka, Japan) operating at 100 kV, 4.8 mA, 9.4 s. The voxel size was not higher than 0.125 mm. The scans were performed according to the manufacturer's instruction and following the principles of radioprotection strictly.

All the images were imported to a dedicated software for dental image visualization and digital planning which is certified for medical use (coDiagnostiX 10, Dental Wings GmbH, Chemnitz, Germany). The visualization was performed on a 34-inch led screen at a resolution of 3,840 x 2,160 pixel. After importing the scans, the images were adjusted to increase the visibility of the anatomical structures of interest, by modifying contrast, brightness, and the magnification ratio.

The images were anonymized fully, by covering personal information of the subjects.

### 2.2. Measurements

**Figure 1:**
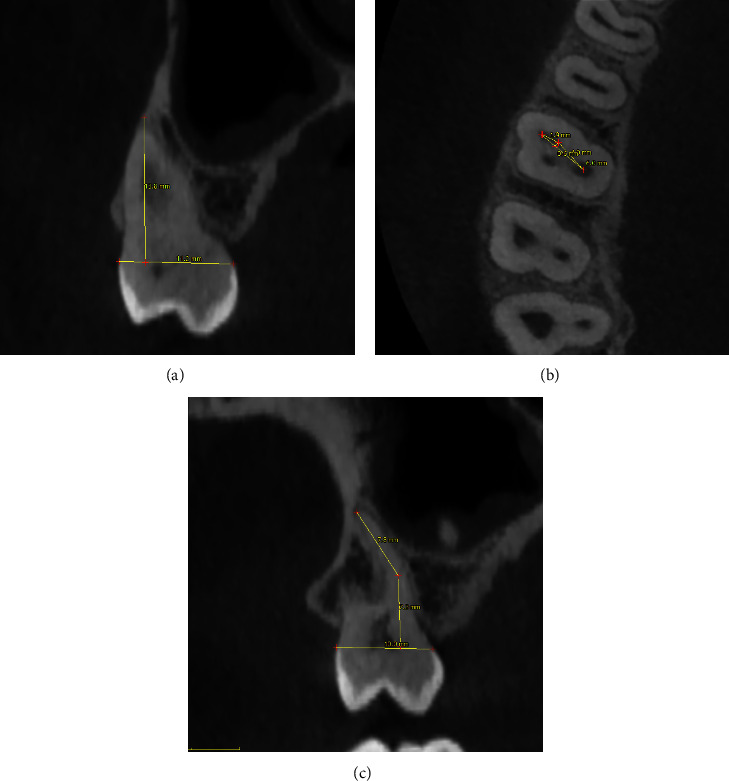
Pictures showing the procedure of performing measures on (a) coronal, (b) axial, and (c) sagittal plane. In (a) the horizontal line is made by linking on the reference plane (coronal in this case) the two points referred to the cemento–enamel junction on buccal and palatal aspect; the vertical line is perpendicular to the horizontal one and directed to the root apex (most apical extent). In (b) the distance between different landmarks is taken on an axial plane, approximately at the level of the floor of the pulp chamber. In (c) the horizontal line is made by linking on the reference plane (sagittal in this case) the two points referred to the cemento–enamel junction on buccal and palatal aspect; the vertical line is perpendicular to the horizontal one and directed to the root apex (most apical extent).

The following measurements were taken, using the appropriate tool, in mm (Figure 1):

- Linear distance between the orifice of the MB2 and the orifice of the palatal canal (P), measured at the level of the floor of the pulp chamber (MB2–P);
- Linear distance between the orifice of the MB2 canal and the orifice of the MB1 canal, measured at the level of the floor of the pulp chamber (MB1–MB2);
- Linear distance between the orifice of the MB1 canal and the orifice of the P, measured at the level of the floor of the pulp chamber (MB1–P);
- Linear distance between the line MB1–P and the MB2 orifice, taken perpendicularly to MB1–P (MB1–P/MB2);
- Length of the mesial root measured both on the coronal and on sagittal plane (*L*c and *L*s);
- Presence of a confluence between MB1 and MB2;
- Vertucci classification of the mesial root anatomy [5].

The measures were taken, following what was done in other previously published studies [16, 17], after detecting the inferior border of the pulpal floor.

Moreover, the age of subjects at the time of radiograph, sex, and voxel size was recorded for each record.

Two operators (IP and MB) performed all the measurements independently, using the same software and the same screen. The two operators were previously calibrated by examining five scans (not included in the study) before the beginning of the study, with a concordance of 90%, considering a tolerance of 0.2 mm in linear measurements.

The data were completely anonymized through the association of each subject to one identification code and the elimination of the document containing the link between them.

### 2.3. Data Analysis

The descriptive analysis was performed by calculating mean, median, standard deviation, and confidence interval for the continuous variables, referred to the measurements. Frequencies were calculated for categorical variables (sex, confluence, and Vertucci classification).

The correlations between the measurements were calculated by means of the Pearson coefficient. Logistic regression analysis served to calculate the influence of the measurements done on the presence of confluence between MB1 and MB2. Moreover, to better understand if a correlation exists between mesial root length and the presence of confluence, the median length was considered as a threshold to establish if roots longer than median are more prone than shorter roots in presenting confluence. Moreover, we tested if *L*s or *L*c could be considered as a predictor for the presence of confluence through receiver operating characteristic (ROC) curve analysis and area under the curve (AUC) calculation. The level of significance was posed to *P* < 0.05. The analyses were all performed by the same operator (SC) with the specific software IBM® SPSS® Statistics version 27.0.1.0 (IBM® Corporation, Armonk, NY, USA).

## 3. Results

**Figure 2:**
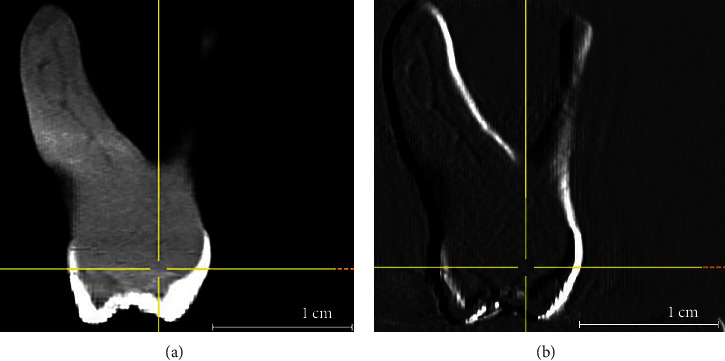
First maxillary molar showing a particular MB root canal anatomy with a 1-1-2 configuration (a). The visualization is enhanced in (b) (courtesy of Dr. Martino Baruffaldi). In (a) the arrow points to the bifurcation of the canals.

The sample was made of 68 Caucasian (Italian) subjects (100 teeth), 55 women, with a mean age of 33.29 ± 15.2 years (median 29.0 years). About root canal anatomy, 30 teeth did not present a confluence of MB1 and MB2, 68 were classified as type II Vertucci, three as type III Vertucci, 22 as type IV Vertucci, and seven as type VI Vertucci.  Figure 2 shows an example of one maxillary molar presenting a particular MB root canal anatomy. Of the total, 41 CBCT scans had 0.080 mm voxel and 59 had 0.125 mm voxel.

The cumulative data of the measurements are presented in Table 1. In Table 2, the results of the correlation analysis are presented. The confluence of MB1 and MB2 was found to be significantly correlated to *L*s (0.203, *P*=0.04).

The regression analysis did not reveal any effect of the parameters evaluated on the presence of confluence of MB1–MB2. An ancillary analysis found that roots with *L*s that exceed the mean value (13.25 mm) are related to an OR = 2.867 (*P*=0.021, 95% CI: 1.176–6.987) of presenting a confluence of MB1–MB2 as compared to roots with *L*s lower than the average value. Considering the 75^th^ percentile (14.56 mm) as *L*s threshold value, roots exceeding this value presented frequent confluence more than three times (OR = 3.635, *P*=0.050, 95% CI: 1.000–13.283). Interestingly, all cases with MB1–MB2 equal or less than 1.40 mm present a confluence (10 cases).

**Figure 3:**
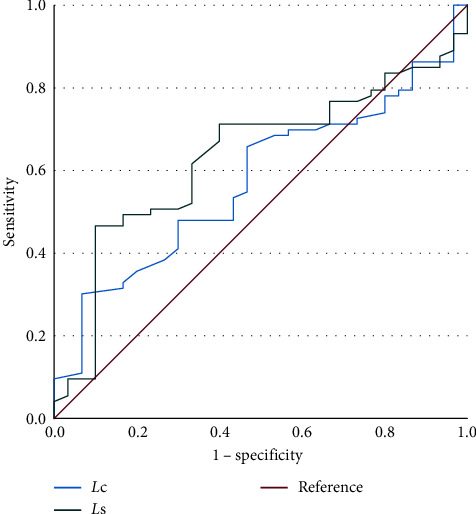
ROC curve. The red line represents insignificant sensitivity of the parameter.

Regarding the ROC curve analysis (Figure 3), the calculated AUC was 0.575 (*P* < 0.05, 95% CI: 0.462–0.689) for *L*c and 0.632 (*P*=0.036, 95% CI: 0.520–0.745) for *L*s.

## 4. Discussion

The present study reported the results about the position of the root canal orifices of maxillary first molars in relation to the characteristics of the MB root, in cases where two MB canals were detected. In general terms, there was a relative heterogeneity of the results in the considered sample. Interestingly, we found a significant correlation between the root length on a sagittal plane and the presence of a confluence between MB1 and MB2, finding the first evidence that the root length, as measured on this plane (for example by using a periapical radiograph), could be an aid for predicting the presence of such confluence.

For the purposes of the study, we performed an evaluation of CBCT images, taken with specific settings that allowed an adequate visualization of the root canal anatomy. The confirmation of the appropriateness of the settings and of the methods used derived from the recently published paper by Mouzinho–Machado et al. [18]. In their research, the authors compared three different voxel sizes (0.08, 0.125, and 0.200 mm) in the scans of 40 maxillary first molars, 20 without MB2 and 20 with MB2. The authors found that the lower the voxel size the higher the accuracy, although the results were considered “good” independently from the setting. Although there were differences between our setting and the experimental, *ex vivo*, one of the studies described, that their conclusions supported our choice, thus also confirming the results of older studies [14, 15]. Other *in vivo* studies used similar settings for the same purposes [19–21]. The use of CBCT, as stated before, is validated in endodontics in all cases requiring a deep investigation of the root canal anatomy both for surgical purposes and for detecting root canal anatomy abnormality of particular characteristics [11, 12]. Although validated, in the present study, we did not perform any radiographic investigation for the purposes of the study, but we examined available records that were anonymized.

The first set of results that deserves a discussion in the light of existing literature is represented by the descriptive values about the root canal orifices. First, the distance between the orifices of the maxillary first molar canals is relatively variable, with a significant range, probably being dependent on the size of the crown and of the tooth in general. The distance MB1–P we found was comparable with those obtained in other studies such as one recent research on Brazilians, which was performed on Micro-CT images [22] and one set in United States, that was made on CBCT scans, taken *in vivo*, as it was done in the present study [17]. Interestingly, the interorifice distance measured and reported by Zhuk et al. [17] is substantially similar to the measures we obtained with the MB1–P distance of 6.87 ± 1.03 mm and MB1–MB2 distance of 2.03 ± 0.55 mm. The same was found on a Korean sample by Lee et al. [23] who examined the location of MB2 relatively to the other root canal orifices and found that the MB1–MB2 distance was 2.10 ± 0.44 mm, coherent with the measures we took. Moreover, the same authors found that the line MB1–MB2 was substantially parallel to the line between the distobuccal orifice and the palatal orifice [23]. The study published in 2021 by Moidu et al. [24] and aimed at examining the association of orifices position and canal configuration in maxillary first molars of a population made of Indians. In all cases that presented MB2, the interorifice distance was about 2.60 mm, without any significant difference with what was found in our study.

We found a statistically significant correlation between the different parameters we measured, and most of the linkage could be explained by a geometric basis, some measures being obviously correlated. As an example, the longer the MB1–P, the longer is the MB2–P, being both related to the bucco-palatal extension of the root chamber. On the basis of this assumption, interestingly we found that the longer the MB1–P, the longer the distance between MB2 and the line MB1–P, revealing an important information for locating the MB2 orifice as related to the other axis [25].

Another important issue we should consider is related to the presence of a confluence between MB1 and MB2. We found a confluence in 70% of the examined teeth, significantly less than the number of cases found by Moidu et al. [24], and this reflected the differences found in the Vertucci configuration. However, in ours and in the study by Moidu et al. [24], Vertucci type II is the most prevalent configuration. Similar to what was found by the same authors, we had the evidence of some correlation between the root length (as measured on a sagittal plane) and the presence of confluence, whose occurrence was more than doubled when *L*s was higher than 13.25 mm. The authors have no scientific basis to explain such evidence although the biological plausibility of such result could find an explanation in the dynamics of root formation; however, more research is needed to explain and support such evidence. We should observe that the accuracy of *L*s parameter in the detection of the confluence (AUC 0.632) could be a first step for validating the use of periapical radiograph alone instead of CBCT for exploring the confluence between MB1 and MB2, but specific studies are needed, comparing the two techniques, to further support such hypothesis, as stated before by other authors [24]. An ancillary analysis revealed that it seemed that there is a threshold of MB1–MB2 (1.40 mm) for being the two canals always confluent, but we do not have enough cases to support such hypothesis.

The results of the study we performed should be weighted considering the limitations of the study design. First, the retrospective nature of the study that created a partial heterogeneity in methods adopted (voxel size); however, the statistical analysis excluded that such factor could have influenced the outcomes. Another issue regards the sample, which is entirely made of Caucasians of Italian origin, with a low mean age. On one side, the considerations we made cannot be extended to all the populations, on the other side we found a substantial similarity to what was obtained by most of the studies with similar purposes. We should further highlight that, as stressed in recently published clinical guidelines, the clinician can be helped in detecting the root canal orifices and, in general, in understanding the root canal anatomy also by means of ultrasonic instrumentation and the use of magnification devices, and not only by using radiographic images as support [26]. Despite such limitations, in our opinion, the present study has the strength of using an adequate voxel size for exploring root canal anatomy, the reliability of the assessment, and the statistical analysis, stratifying the available data.

Reading the results, we can conclude that the position of MB2 as related to MB1 and P orifices could be estimated based on of the position of the other anatomical landmarks. The presence of confluence between MB1 and MB2 was minimally correlated to root length as evaluated on a sagittal plane and to the distance between MB1 and MB2 orifice. This result, although it needs more scientific support, could be of help for the clinician who can approach the treatment without the need of performing a CBCT, by using the bidimensional radiograph to have an estimate of the possibility of having MB1 and MB2 confluent.

More studies are desirable to better investigate, on larger samples, the correlation between different anatomical parameters in maxillary molars, thus giving more information to the clinician before approaching endodontic treatment of these teeth.

## Figures and Tables

**Table 1:** Measurements of the interorifice distance.

Parameter	Mean ± SD (mm)	Median (mm)	Range (mm)
MB1–P	5.93 ± 0.82	5.91	4.30–8.10
MB1–MB2	2.41 ± 0.66	2.40	0.80–3.70
MB2–P	3.71 ± 0.80	3.70	1.80–5.60
MB1–P/MB2	0.74 ± 0.27	0.72	0.00–1.60
*L*c (coronal)	12.15 ± 1.64	12.18	8.60–16.70
*L*s (sagittal)	13.25 ± 1.78	13.28	9.90–16.80

**Table 2:** Table of correlations: Spearman's P.

		MB2–P	MB1–MB2	MB1–P	MB1–P/MB2	*L*c (coronal)	*L*s (sagittal)
MB2–P	Corr.	—	—	—	—	—	—
	Sign.	—	—	—	—	—	—

MB1–MB2	Corr.	−0.372	—	—	—	—	—
	Sign.	<0.001	—	—	—	—	—

MB1–P	Corr.	0.659	0.403	—	—	—	—
	Sign.	<0.001	<0.001	—	—	—	—

MB1–P/MB2	Corr.	0.120	0.336	0.274	—	—	—
	Sign.	0.228	<0.001	0.005	—	—	—

*L*c (coronal)	Corr.	−0.039	0.223	0.133	0.118	—	—
	Sign.	0.698	0.023	0.180	0.234	—	—

*L*s (sagittal)	Corr.	−0.087	0.169	0.093	−0.039	0.760	—
	Sign.	0.384	0.87	0.350	0.696	<0.001	—

## Data Availability

Data will be available on request.
